# Impact of rapid genomic testing on clinical outcomes of acutely unwell children presenting with severe epilepsy

**DOI:** 10.1038/s41431-025-01870-5

**Published:** 2025-05-21

**Authors:** Erina Sasaki, Philip Millington, Taisiia Sazonova, Lucy Hanington, Andrew Parrish, Benito Banos-Pinero, Helen Lord, John Taylor, Ramanand Jeeneea, Charlotte Sherlaw-Sturrock, Amitav Parida, Julie Vogt, Swathi Naik, Mario Sa, Usha Kini

**Affiliations:** 1 Oxford Centre for Genomic Medicine, Oxford University Hospitals NHS Foundation Trust, Oxford, UK; 2 Department of Paediatric Neurology, Oxford University Hospitals NHS Foundation Trust, Oxford, UK; 3 Radcliffe Department of Medicine, Nuffield Department of Clinical Laboratory Sciences, University of Oxford, Oxford, UK; 4 Royal Devon University Healthcare NHS Foundation Trust, Exeter, UK; 5 Oxford Regional Genetics Laboratories, Oxford University Hospitals NHS Foundation Trust, Oxford, UK; 6 Clinical Genetics Department, Birmingham Women’s Hospital NHS Foundation Trust, Birmingham, UK; 7 Department of Paediatric Neurology, Birmingham Women’s Hospital NHS Foundation Trust, Birmingham, UK

**Keywords:** Epilepsy, Paediatrics

## Abstract

About 30% of epilepsy patients remain unresponsive to standard antiseizure treatment. Increasing evidence suggests that genetic epilepsies may respond better to targeted management. In this study, we therefore evaluate the therapeutic benefits of rapid genetic testing in children with severe epilepsy. Methods: the clinical data of patients with epilepsy referred for rapid whole-exome sequencing were systematically collected at two large paediatric/neurogenetic centres (Birmingham/Oxford) in the United Kingdom over 3 years (2019–2022), with follow-up at 12 months post-diagnosis. The demographics, diagnostic yield, management by gene function and seizure group (SZ-seizures only or SZ+ seizures with co-morbidities) were explored. Results: among the 106 eligible patients, the age at testing ranged from 0 to 16 years with a median of 7 months. Underserved ethnic groups, e.g., British Asians and Black British, were well-represented. Thirty-nine genes affecting 49 patients were identified, giving an overall diagnostic yield of 46%, which was further enhanced to 51% (31/61) in the SZ+ group. Twenty percent of genes identified affect ion channels and patients were more likely to present early (<6 months old) and respond to a gene-directed treatment (*p* = 0.004483). Seizures secondary to metabolic disorders responded to bespoke therapy. A fifth (22/106) of tested patients and 45% (22/49) of those diagnosed had their management impacted. At the 12-month follow-up, 9/15 (60%) patients remained seizure-free following gene-targeted management. Conclusion: this study demonstrates high diagnostic yield and significant therapeutic benefit from rapid genetic testing in patients with epilepsy. The gene function categories were statistically significant predictors of management change.

## Introduction

Epilepsy is a common chronic neurological disorder affecting one in 107 people in the United Kingdom (UK), with an estimated 112,000 of these being children and adolescents [1]. A high rate of morbidity and mortality is associated with this disorder, with ~30% of patients remaining unresponsive to standard antiseizure medication (ASM) [2–6]. The current treatment strategy for epilepsy, including the choice of ASM, is guided by factors such as seizure type, age of patient, and patient choice [7]. Paediatric epilepsies have diverse aetiologies and clinical outcomes and can occur secondary to a brain insult such as infection, trauma, tumours, and autoimmune disorders. However, in those with idiopathic epilepsy, a monogenic cause is strongly suspected, with some reports suggesting that more than 50% of cases may have an underlying genetic cause [6, 8]. The likelihood of identifying a genetic cause appears to be higher in severe early-onset epilepsy, regardless of whether the seizures occur in isolation or together with other co-morbidities (syndromic epilepsy) [6, 8]. Some recent studies of patients with genetic epilepsies have highlighted the importance of the genetic diagnosis in guiding the management [6, 8–10].

Genetic epilepsies may occur due to a chromosomal aberration (copy number variant or CNV) or a variant in a single gene (monogenic disorder). Some chromosomal loci that have been linked to seizures include chromosome 2q24.3, 7q11.23, 15q11-q13, 22q13.3 [11]. Over a thousand genes known to be associated with epilepsy have been identified but current National Health Service (NHS) epilepsy gene panel (Reference number 59, R59) includes over 600 carefully curated genes where robust scientific evidence for their pathogenicity exists—the most common mutated genes being *PRRT2, SCN1A, KCNQ2* and *SLC2A1* [12].

With the advances in genomics testing technology and a reduction in the cost of testing, exome/genome sequencing has become available as a first-line investigation. Reference number 14 (R14) rapid whole exome sequencing (WES) [13], a United Kingdom Accredited Service (UKAS) [14], became available in October 2019 as an urgent test for acutely unwell children with a suspected monogenic cause where the genetic test result has the potential to influence the management of patients. Since October 2022, the testing strategy has been changed to rapid whole genome sequencing (WGS).

The main aim of our study is to assess the diagnostic yield and clinical benefits from rapid WES testing in a cohort of patients presenting acutely unwell with seizures at two large genetics/paediatric neurology centres in the UK. Furthermore, the therapeutic management of these patients in the context of the gene function is explored.

## Methods

Medical records of consecutive patients presenting acutely unwell with a likely underlying monogenic cause for their seizures and undergoing rapid WES, over a period of 3 years (between October 2019 and September 2022), in tertiary centres at Birmingham Women’s and Children’s Hospital and Oxford University Hospitals, were methodically accessed. These centres cover an ethnically diverse population of 8 million [15]. The eligibility criteria for rapid WES for patients with seizures included: early onset seizures (<2 years of age), severe intractable epilepsy, and/or seizures resulting in intensive care admissions (including status epilepticus). All families were consented for testing using standard record of discussion forms. Patients were identified from a prospectively maintained departmental rapid WES database at both centres.

Both phenotype and genotype data were reviewed. Specifically, information was gathered about indication for rapid WES, sex, ethnicity, age at testing, age at diagnosis, additional co-morbidities, genomic variant/s identified, and changes in management resulting from the diagnosis. Follow-up clinical data at 12 months post-diagnosis were gathered for those who had changes to their management. Specific follow-up data included frequency of seizures, number of attendances at an emergency department, admission to the intensive care unit and mortality.

The rapid WES service uses a gene agnostic approach and analyses the coding regions and splice sites of 23,244 genes by next-generation sequencing (Twist Core Human Exome/Illumina NextSeq/NovaSeq) [13]. The variants were classified using ACMG guidelines [16]. The overall sensitivity for detecting heterozygous small variants is >99%, with a read depth coverage of >20. We recorded the effect of variants on protein, such as loss or gain of function, based on in-silico tools, meta-predictor scores, or functional study where available. Advertised turnaround time (TAT) is 2–3 weeks. Trio (proband and parents) sequencing is preferred but duo and singleton samples are also accepted where parental samples are unavailable.

We classified our cohort into 2 main groups, Group SZ—seizures only, and Group SZ+-seizures associated with other co-morbidities (such as developmental delay, regression, learning difficulties, behavioural problems, congenital malformation/s, and dysmorphism), to study the characteristics of genetic epilepsies. We also classified the causal genes identified by their biological function and studied their response to therapy. We also studied the inheritance patterns by ethnicity.

### Statistical analysis

Chi-square test was used to compare the changes in management between the SZ and SZ+ groups and Pearson’s Chi-square test was used to compare the changes in management by gene. Fisher’s exact test was conducted to analyse the association between ethnicity and inheritance patterns.

## Results

In the 3 years of rapid WES service, ‘seizure’ was the most common neurological indication for requesting rapid WES. A total of 107 such cases were referred; one family declined genetic testing and have been excluded from the analysis. Full details are summarised in the Supplementary Material.

### Demographics

Forty-four patients (42%) were female and 62 (58%) were male. The average and median ages at the time of requesting rapid WES was 1145 days (3 years) and 221 days (7 months), respectively, with a range of 0–5897 days (16 years). Seventy-seven percent (82 of 106) received the results within the expected TAT of 21 days. Of the remaining 24 patients, the majority received the result within 30 days, with an average TAT of 26 days; one patient was an outlier with a TAT of 191 days—in most cases the prolonged TAT was due to a delay in receiving parental samples for testing.

**Fig. 1:**
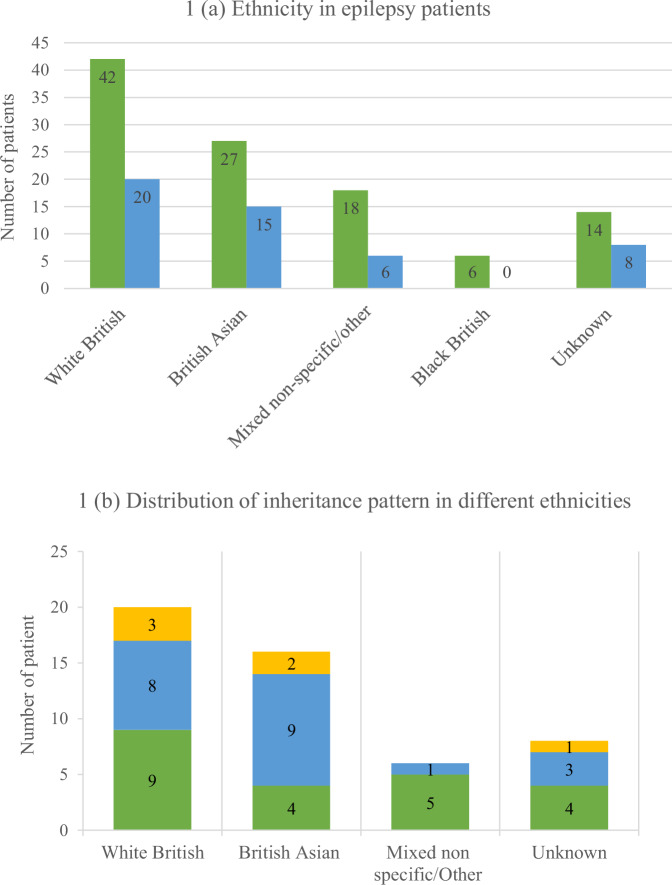
Ethnicity and inheritance patterns in patients tested. **a** Distribution of Ethnicity in patients who underwent R14 and in those with a molecular diagnosis. Green bar: number of patients who underwent R14 in each ethnic group. Light blue bar: number of patients where molecular diagnosis was reached in each ethnic group. **b** Breakdown of inheritance patterns by ethnicity. Green bar indicates AD, Autosomal dominant; Blue bar indicates AR, Autosomal recessive; Yellow bar indicates X-linked condition.

We used the ethnicity data to assess the uptake of testing amongst diverse ethnic groups and noted that the Asian and Black ethnic minorities were well-represented (Fig. 1a). The inheritance patterns across ethnicities, showed that amongst 15 British Asian patients with a diagnosis, 9 (60%) were diagnosed with an autosomal recessive disorder and all of them were found to carry homozygous variants reflecting consanguinity. No genetic diagnosis was made in the Black ethnic minority group.

### Co-morbidity

The most common co-morbidity reported in SZ+ group of 64 patients was neurodevelopmental delay (21, 33%), followed by regression (14, 23%) and congenital brain malformation (10, 17%). Other co-modibities were hypotonia (9, 14%), dysmorphic features (8, 12%), dystonia (5, 8%), ataxia (5, 8%), nystagmus (3, 5%), congenital diagphragmatic hernia (1, 1.5%), and scoliosis (1, 1.5%).

### Diagnostic yield

Fifty-two of 106 (49%) patients were found to have pathogenic or likely pathogenic variant/s. Of these, in five cases an initially identified variant of unknown significance (VUS) was reclassified as ‘likely pathogenic’ following further investigations which included biochemical testing, trial of bespoke therapy, RNA studies, parental testing and segregation studies. In those without a molecular diagnosis, there were a further 8 cases with VUS, and 3 had incidental findings which did not explain their seizures (*DMD, G6PD*, 2p21 deletion); in 45 cases no reportable variants were identified. Nearly half the patients (24 of 49, 49%) were less than 6 months old at the time of diagnosis. Overall, 21 of 49 (43%) patients were diagnosed with an autosomal recessive disorder, 22 (45%) with an autosomal dominant disorder and 6 (12%) with an X-linked disorder.

### Comparison between SZ and SZ+ groups

**Fig. 2:**
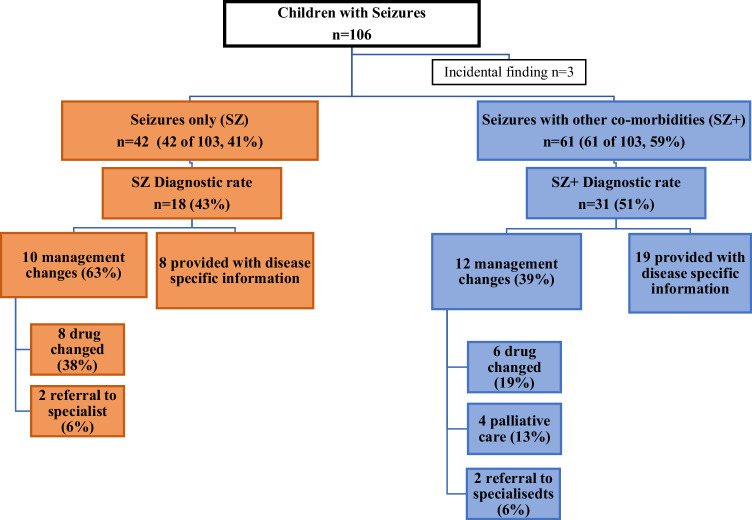
Comparison of diagnostic yield and change in management in patients with seizures only (SZ) vs seizures with other co-morbidities (SZ+).

Of the total 106 patients, 42 patients (41%) were in the SZ group and 61 (59%) were in the SZ+ group. Details of the diagnostic rate and management change in these two groups are summarised in Fig. 2.

Sixty-two percent of cases in SZ+ group were analysed as a trio. Thirty-one of 61 patients (51%) in the SZ+ group had a confirmed molecular diagnosis, and in 12 of 31 (39%), the diagnosis resulted in active changes to the clinical management. Six patients (19%) either had their medication changed or had a new medication added. Two (6%) were referred to specialists for further management (ophthalmology, endocrinology, and neurometabolic department) and, after careful consideration of all relevant information, four (13%) patients were referred for palliative care.

Ninety percent of cases in SZ group were analysed as a trio. Eighteen of 42 patients (43%) received a molecular diagnosis and more than half of these patients (10 of 18, 56%) had their management changed. Seven patients whose medication was changed or had a new medication added were all found to carry a variant in ion channel genes (two *SCN1A* LoF variants, two *SCN2A* gain-of-function variants, one *SCN8A* gain-of-function variant, and two *KCNQ2* LoF variant). Additionally, empiric treatment of pyridoxine, folinic acid, biotin was withdrawn in one following identification of a chromosome 2q34.3 duplication (including *SCN1A, SCN2A*, and *SCN3A)*. In one patient (*POLG*) sodium valproate was withdrawn [17]. One patient (*SLC6A5*) was commenced on clonazepam for gene-related hyperekplexia and referred to specialist neurologist for advice [18]. No SZ patients were directed to palliative management.

In the SZ+ cohort, more than half (17 of 31, 55%) of the diagnoses made were monogenic autosomal recessive syndromic epileptic disorders and 13 (72%) of these were due to biparental, homozygous inheritance. In contrast, the majority (13 of 18, 72%) of SZ patients were diagnosed with autosomal dominant conditions and 9 (9 of 13, 75%) of them were found to carry a variant in ion channel genes*: SCN1A, SCN2A, SCN8A, KCNQ2, KCNT1*. In five of these patients the disease-causing variant (*SCN1A*, *SCN2A, KCNQ2*, and *KCNT1)* was inherited from a parent (but only two parents were reported to be affected).

### Genetic variants and clinical impact

A total of 49 pathogenic/ likely pathogenic (P/LP) variants were identified in 39 genes and 4 CNVs. The management changes were assessed, excluding cases with incidental findings. Disease-specific information and advice were given to patients with incidental findings. CNVs included 5p15 duplication syndrome, a complex 9p24 deletion/9p24 duplication/12q24 duplication encompassing *SMARCA2* and *RFX3* in the deleted region and *SETD1B* in the duplicated region, 3q28q29 microduplication encompassing exon 1 to 4 of *FGF12*, 2q24.3 duplication encompassing *SCN1A, SCN2A*, and *SCN3A*. One patient was found to have 2p21 homozygous deletion encompassing *SLC3A1* and *PREPL* that do not explain the seizures. We broadly categorised genes contributing to epilepsy into 4 groups based on their biological functions: genes affecting (i) ion channels, (ii) metabolic pathways, (iii) embryonic brain development, and (iv) miscellaneous (including mitochondrial genes). We studied the distribution of these in the 2 seizure groups; findings are summarised in Table 1.

**Table 1:** Different affected genes by seizure group.

Group of genes	Ion channel	Metabolic	Developmental		Miscellaneous/Mitochondrial
SZ (*N* = 18)	*KCNT1*	*B4GALNT1*	*CDKL5*		*DNM1L*
	***KCNQ2*** *(N* = *3)*		*CYFIP2*		***POLG***
	*PRRT2*		***SLC6A5***		
	***SCN1A*** *(N* = *2)*		*SLC13A5*		
	***SCN2A*** *(N* = *2)*				
	***SCN8A***				
	**2q24 dup**				
SZ+ (*N* = 31)	*CACNA1E*	***ACBD5***	*ANKRD11*	*STXBP1 (N* = *2)*	*BSND*
	***KCTD7***	***ALG13***	*ARX*	*TBCK*	*FLVCR2*
	***KCNQ5***	***ASNS***	***BRAT1***	**3q28q29 dup**	***GLDC***
		***CAD***	*CUL4B*	5p15 dup	*HADHB*
		*MOCS2*	*DNM1*	9p24del&dup/12q24 dup	*MFSD8*
		***SLC52A3***	*MECP2 (N* = *2)*		*STUB1*
		***SUOX***	*PRUNE1*		
		***TPP1***			

*N* refers to the number of patients affected. For those genes where no number is indicated in brackets, there was only a single patient identified. Genes in bold indicate those with management change. SZ Seizures only; SZ+ Seizures with other co-morbidity.

A total of 13 patients had a P/LP variant in the ion channel genes and 10 of these patients had a drug change to their management; this was usually changed to another more effective ASM directed by the genotype. Nine genes causing a metabolic disorder were identified and treatment was changed for 7 patients: 2 were considered for conservative management and the remaining patients had a more targeted bespoke therapy such as oral uridine for *CAD*, cerliponase alfa for *TPP1*, riboflavin supplementation for *SLC52A3*, and ketogenic diet for *ALG13* variants [19–22]. No drug changes were made for patients with variants in developmental genes, however one was referred to a specialist service (*SLC6A5*) and the other for palliative care (*BRAT1*). Two genes affecting mitochondrial function were also identified: valproate was avoided for the *POLG-*related seizures [17], and the *GLDC* patient was referred for palliative care. For all cases referred for palliative care the genetic diagnosis supported the clinical decision, based on the reported poor prognosis associated with those disorders. Table 2 summarises the management changes, by genotype, in each seizure group.

**Table 2:** Summary of age at diagnosis, details of variants identified and management change in SZ/SZ+ groups.

Group	Age at diagnosis	Gene affected	Gene function	Class	Variant type	Change in management	Outcome at 12 months after change in management
***SZ***	25 days	*KCNQ2*	IC	5	LoF	Commenced on phenobarbitone	Seizures stopped. Medications weaned off.
	29 days	*KCNQ2*	IC	5	LoF	Commenced on phenobarbitone	Only 1 minor seizure. Medication weaned off.
	38 days	*SCN2A*	IC	5	GoF	Changed to carbamazepine	Seizures stopped. Medication weaned off.
	40 days	*SLC6A5*	Dev	5	LoF	Clonazepam added. Specialist (neurology) referral	Seizures stopped. Medications weaned off.
	58 days	*SCN8A*	IC	5	GoF	Changed to sodium channel blocker (lamotrigine) and nitrazepam	Seizures continued (daily multiple seizures). Two attendances to A&E department.
	102 days	2q24.3 dup	CNV	5	n/a	Changed to carbamazepine	Seizures stopped. Medication weaned off.
	152 days	*SCN2A*	IC	4	GoF	Changed to carbamazepine	Lost to follow-up
	392 days	*SCN1A*	IC	4	LoF	Changed to sodium valproate	Seizures continue. At least one admission to PICU required.
	16 months	*POLG*	Misc/Mito	5	LoF	Ketogenic diet added Avoidance of sodium valproate. Specialist (mitochondrial service) referral	Deceased
	19 months	*SCN1A*	IC	4	LoF	Changed to sodium valproate	Seizures only at the time of viral illnesses. Continued on medication.
**SZ**+	20 days	*ASNS*	Met	5	LoF	Palliative care	Deceased
	23 days	*BRAT1*	Dev	5	LoF	Palliative care	Deceased
	29 days	*GLDC*	Misc/Mito	5	LoF	Palliative care	Deceased
	120 days	*CAD*	Met	5	LoF	Changed to uridine	Seizures stopped. Under the care of a metabolic specialist.
	172 days	*SUOX*	Met	5	LoF	Palliative care	Deceased
	272 days	*SLC52A3*	Dev	4	LoF	Changed to riboflavin	Seizures stopped. One PICU admission and long-term inpatient care required.
	1 year	*ALG13*	Met	5	LoF	Ketogenic diet added	Seizures increased in frequency: absence seizures most days and myoclonic jerks up to 2-3 times a day. Three attendances to A&E department.
	2 years	*KCTD7*	IC	5	LoF	Specialist (metabolic medicine) referral	Seizures stopped. Continued only on medication for dystonia. One attendance to A&E department.
	2.4 years	*ABCD5*	Met	5	LoF	Specialist (metabolic medicine) referral	Seizures increased. Required several attendances to A&E department.
	4.5 years	*TPP1*	Met	5	LoF	Commenced on ceroliponase, specialist (metabolic medicine) referral	Seizures continued but at reduced frequency (50%). Successfully weaned off topiramide and clobazam.
	7 years	*KCNQ5*	IC	4	GoF	Changed to carbamazepine, ketogenic diet added	Seizures continued. A couple of Tonic-clonic seizures a day. Clobazam added. Attended A&E department 4 times.
	13.5 years	3q28q29 del 3q28dup, 10qdup	CNV	5	n/a	Changed to phenytoin	Lost to follow-up

4, class 4 likely pathogenic variant; 5, class 5 pathogenic variant.

*CNV* copy number variants, *del* deletion, *Dev* developmental genes, *dup* duplication, *GoF* gain-of-function variant, *IC* ion channel, *LoF* loss-of-function variant, *Met* metabolic genes, *Misc/Mito* miscellaneous/mitochondrial genes (both nuclear and mitochondrial genes).

**Fig. 3:**
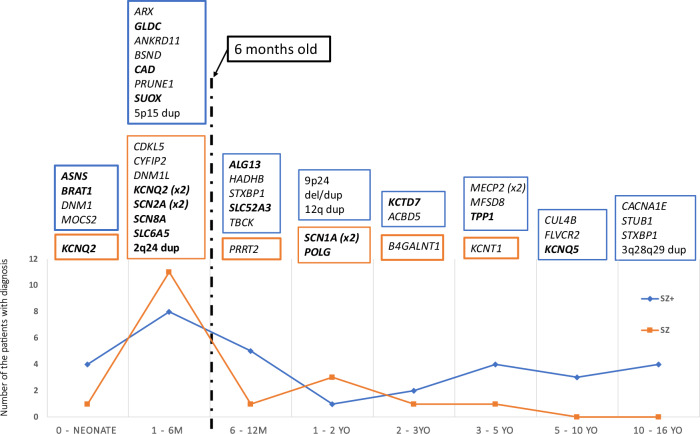
Distribution of affected genes by age and seizure group. Genes associated with active management change are highlighted in bold. *X*-axis indicates age at diagnosis and *Y*-axis indicates the number of patients. Blue Box/line indicates SZ+. Orange Box/line indicates SZ patients. Vertical broken line shows the 6-months mark.

We also studied the distribution of the genetic variants by age at diagnosis and seizure group—described in Fig. 3. Twelve of 31 patients (39%) in the SZ+ group were diagnosed at or before the age of 6 months, but this did not result in a change in management for any of them. In the SZ group, 11 out of 18 received a molecular diagnosis before the age of 6 months with 8 of them (73%) receiving a change in their management.

### Clinical outcomes at 12 months

**Fig. 4:**
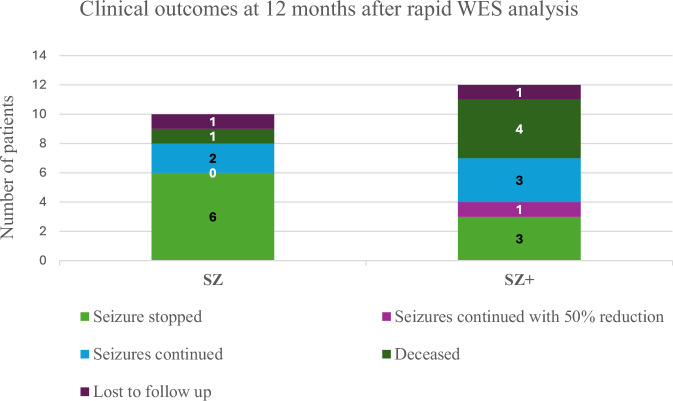
Clinical outcomes of 10 SZ patients and 12 SZ+ patients at 12 months following gene-targeted management. The light green bar indicates seizure stopped; the light purple bar indicates seizures continued with a 50% reduction; the light blue bar indicates seizures continued; the dark green and purple bar shows the number deceased and lost to follow-up, respectively.

We assessed the clinical outcomes at 12 months post-diagnosis of those 22 patients whose clinical management was altered after reaching a genetic diagnosis and summarised in Table 2. Five patients were deceased: 3 mitochondrial (*BRAT1, GLDC, POLG*) and 2 metabolic (*ASNS, SUOX*). All these patients (except for the patient with the *POLG* variant) had been directed to palliative care following the diagnosis. Two of the 22 patients were lost to follow up in clinic. Of 15 patients whose clinical data were available, 9 (60%) were seizure-free at 12 months following the introduction of the gene-directed treatment. These included six ion channelopathy patients (*KCNQ2, KCTD7, SCN1A, SCN2A*, and 2q34.3 duplication), one patient with a variant in a developmental gene (*SLC6A5*), and 2 metabolic patients (*CAD* and *SLC52A3*) who received bespoke therapy. A 50% reduction in seizures at 12 months following commencement of the gene-directed treatment was seen in one patient (*TPP1*). Three (20%) patients (*KCNQ5, SCN1A, SCN8A*) showed no improvement in frequency of seizures on the targeted treatment. Four patients (*ABCD5, ALG13, KCNQ5, SCN8A*) attended an emergency department at least once in the 12-month period for seizure-related problems. One patient (*KCTD7*) required six attendances to an emergency department not due to epilepsy but due to underlying dystonia. Two patients (*SCN1A, SLC52A3*) required at least one admission to PICU followed by long term inpatient management. Clinical outcomes at 12 months by seizure group is summarised in Fig. 4.

### Statistical analysis

We compared the changes in management between the SZ and SZ+ groups using the Chi-square test. No significant association (*χ*² = 0.4104, df = 1, *p* = 0.5218) was identified. Using Pearson’s Chi-squared test, we compared the changes in management by gene function and noted a significant association (*χ*² = 13.072, df = 3, *p* = 0.004483), particularly for the ion channel and the developmental gene groups. Fisher’s exact test was conducted to analyse the association between ethnicity and inheritance patterns; no significant correlation was observed (*p* = 0.2749).

## Discussion

Our study assessed the diagnostic yield and therapeutic benefits of rapid exome sequencing in a cohort of 106 consecutive patients with an acute seizure presentation. The overall diagnostic yield in our cohort is 47%, which is slightly higher than the previously reported figure (40%) for all rapid WES cases (seizure and non-seizure) [23]. The diagnostic yield is further enhanced in patients with syndromic epilepsy (51%), compared to those with isolated seizures.

The results of this study confirm the high level of genetic heterogeneity seen in epilepsy—39 genes and 5 copy number variants affecting 49 patients. Most patients (60%) were diagnosed in infancy (<1 year of age), with those <6 months old having the highest diagnostic yield. Those with an ion channelopathy were more likely to present early in life (<6 months of age) with isolated seizures. However, these genes are known to cause developmental delay and intellectual disability, and it is likely that these features were not reported in our cohort because of the early presentation and diagnosis. In this cohort, the most common gene affected was *KCNQ2*, noted in three patients aged between 3 and 7 weeks old. This was followed by *SCN1A* and *SCN2A* where there were two patients affected for each gene. These findings are in keeping with recent reports [6]. None of the three patients with *SCN1A* variants had a clinical diagnosis of Dravet syndrome: two were seizure-free at 12 months (one of who was diagnosed with Genetic Epilepsy with Febrile Seizures Plus (GEFS+)), while the third continued to have seizures. *KCNQ2* loss-of-function (LoF) variants are associated with self-limiting neonatal epilepsy, while variants resulting in dominant-negative effects cause severe neonatal-onset developmental and epileptic encephalopathy [19, 24, 25]. As expected, both patients with a *KCNQ2* LoF variant and *SLC6A5* variant were seizure-free at the 12-month follow-up. *KCTD7* encodes a member of the potassium channel tetramerization protein and pathogenic variants in this gene cause neurodegenerative disorders ranging from early onset intractable myoclonic epilepsy and developmental regression to neuronal ceroid lipofuscinosis [20]. Although its direct effect on potassium channel remains to be elucidated, its clinical features overlap that of ion channelopathy and metabolic disorder [21, 22], we included this gene in the ion channel category. Our patient’s seizures have stopped at the 12-month follow-up, but some movement abnormalities were persisting. Most patients with ion channelopathy responded well to a change in the ASM to a more effective targeted ASM, and there was no mortality within this group, indicating that ion channel related epilepsies may have a better prognosis.

In the metabolic disorder group, although most patients had a change in their management, this was in the form of bespoke therapy to reverse the enzymatic defect rather than a change in ASM [26–29]. The fact that standard ASM therapy is less helpful in controlling the epilepsy in these patients emphasises the importance of early and rapid genetic testing. These patients all presented with associated co-morbidities and all except one were diagnosed before the age of 1 year. Of note, many seizure-related genes identified in this study (for e.g. *ACBD5, SLC6A5, STUB1*) are not included in the routine epilepsy panel (R59) and this highlights the benefit of the gene-agnostic analytic approach offered by the rapid WES testing.

The largest group of genes (40%) was that affecting brain development by a direct effect on basic cellular processes and functions, with the most common genes affected being *MECP2* and *STXBP1*, where there were two patients per each affected gene. Three of these patients had a later age at diagnosis (3–16 years), but this may merely reflect the fact that rapid WES testing was not routinely available in clinical practice prior to October 2019. This group of genes was not amenable to a change in management. It is well known that many of these developmental genes cause drug-resistant epilepsy, highlighting the unmet clinical need for effective therapy in this group. Prioritising these disorders for the development of future molecular therapy is therefore vital.

Two genes affecting mitochondrial function (*POLG* and *GLDC*) were identified in this cohort. In both cases, there was prior clinical suspicion of a mitochondrial disorder and mitochondrial DNA analysis had been commenced in parallel to the rapid WES. While the *POLG* variant was managed by withdrawing valproate, the *GLDC* patient was referred to palliative care. In total, four patients were referred for palliative care based on their clinical presentation and poor prognosis as predicted by the genetic diagnosis. An early genetic diagnosis is therefore helpful not only in directing a change in medication but also in determining the most suitable level of care.

According to the Office for National Statistics, the total UK population (England and Wales) of 60 million breaks down to 82% White British, 10% British Asian, 4% Black British, and 5% mixed and other ethnicities [30]. In our study, 25% were British Asians and 6% were Black British, thereby demonstrating the uptake of genetic testing across diverse and even under-served ethnic groups. No genetic diagnosis was made in the Black British group suggesting that more research needs to be done in this ethnic group, in particular exploring genetic variants that may be ethnicity-specific. Although the most common inheritance pattern seen in White British patients was autosomal dominant (45%), and autosomal recessive (due to consanguinity) in British Asians, a large proportion of White British patients (40%) also had an autosomal recessive disorder and similarly 40% of British Asians had an autosomal dominant/X-liked disorder. This again emphasises the importance of a gene-agnostic approach in the analysis of exome sequencing data to eliminate the assumed bias of inheritance patterns based on ethnicity.

Overall, a significant number (21%) of paediatric neurology patients with seizures had an impact on their management following rapid genetic testing, with 9 out of 15 patients showing a sustained response to targeted treatment at 12 months follow-up. Although they ranged in age from neonates to adolescents, most of them were diagnosed and treated in the first 6 months of life. Our study therefore demonstrates that rapid genetic testing allows the institution of early targeted treatment with an aim to improve the clinical outcome. This was particularly notable in the metabolic group where standard antiepileptic drugs were ineffective. Reaching a diagnosis in nearly half of our patients enabled clinicians to provide the families with disease-specific information such as natural course of disease, prognosis and management, identification of at-risk family members, provision of accurate recurrence risk advice and access to relevant prenatal testing and reproductive options.

Although our diagnostic yield was sufficiently high to have a significant impact on clinical management, about half of the cases remained unsolved. The two main causes for this may be that some patients may have a variant in a novel gene not yet known to cause human disease or that current testing strategies and pipelines are unable to identify rarer mechanisms of disease causation. The availability of parental samples in the duo and singleton referrals may have enhanced the diagnostic rate further.

One of the main limitations of our study is the small sample size despite the inclusion of consecutive patients presenting acutely unwell, over a 3-year period, across wo large regional UK centres. To understand the true impact of this testing, a much larger cohort needs be recruited and longer-term follow-up of these patients with health economics cost analysis should be carried out. With an escalation in clinician awareness of the benefits and accessibility of rapid genomic testing in the UK, repeating the study in a new cohort of patients receiving WGS since October 2022 is likely to be valuable in terms of assessing the diagnostic yield and the change in management based on genome sequencing results. Additionally, patients with severe epilepsy would benefit from ultra-rapid WGS to institute the most appropriate treatment at the earliest time possible, with a view to reducing the impact on the developing brain in young children from severe seizures.

## Availability of data and materials

All data generated or analysed during this study are included in the manuscript and its Supplementary Files.

## Supplementary information

Supplementary clinical and genomic data
